# The Differentiation of True Dysentery Bacilli from Allied Species

**Published:** 1918-05

**Authors:** 


***The Differentiation of True Dysentery Bacilli from Allied Species.*** By F. W. Andrewes, Major R. A. M. C. (T.), Professor of Pathology in the University of London. Abs. from the *Lancet*, April 20, 1918.

The author examined upwards of a hundred strains of atypical dysentery bacilli. Owing to difficulty in obtaining rabbits he was unable to investigate the pathogenicity of all the strains, and for the same reason could only employ the stock sera in general use. To the *true dysentery* bacilli, viz. the Shiga and Flexner (including Flexner-Y) groups, he thinks a third should probably be added, i. e. the " Strong " type. He groups the atypical dysentery bacilli which are liable to be confounded with genuine dysentery bacilli into three classes; — for which he suggests the following names.

1. *Bacillus ambiguus.* An organism resembling Shiga's bacillus, but forming indol and otherwise separable from Shiga. It has no demonstrable relation to human disease.

2. *Bacillus alkalescens.* Resembling Flexner's bacillus, but fermenting dulcite and producing alkali with much vigor. It appears quite non-pathogenic.

3. *Bacillus dispar.* This term is suggested as a convenient one for the lactose-fermenting organisms of the dysentery group, which are liable to be confounded with Flexner's bacillus. Some members of this group are decidedly pathogenic for the rabbit.

Of the tests used the acid agglutination test seems to be of great practical value; it does what no one specific serum can, and the margin of error is perhaps less than when even a number of specific sera are used. The author has tried it upon a large quantity of strains of Shiga and Flexner-Y on the one hand, and B. ambiguus, alkalescens, dispar, and other miscellaneous organisms, on the other, and agrees with Michaelis that in the presence of a trace of protein the dysentery bacilli may be readily distinguished from their allies. The method is as follows :

Six stock solutions are made up with normal soda and normal acetic in the following proportions :

| | Normal NaOH | Normal acetic acid | Distilled water |
|---|---|---|---|
| N° 1. . . . . | 5 cc. | 7.5 cc. | 87.5 cc. |
| N° 2. . . . . | 5 " | 10.0 " | 85.0 " |
| N° 3. . . . . | 5 " | 15.0 " | 80.0 " |
| N° 4. . . . . | 5 " | 25.0 " | 70.0 " |
| N° 5. . . . . | 5 " | 45.0 " | 50.0 " |
| N° 6. . . . . | 5 " | 85.0 " | 10.0 " |

affording a constant strength of sodium acetate with an excess of acetic acid ranging from 1/40 N in N° 1 to 4/5 N in N° 6. For the test a 24-hour agar culture of the bacillus is emulsified in 20 cc. of distilled water; broth cultures cannot be used on account of the sodium chloride present. Three parts of emulsion are measured into each of six test-tubes or agglutination tubes, with the addition to each tube of a little protein (for a small test-tube holding 1 or 2 cc. a drop of a tenfold dilution of normal human serum suffices). One part of each stock acid solution is now added to the six tubes respectively and well mixed. The tubes are incubated for two hours at 37° C. and the results read with the naked eye, preferably after standing at laboratory temperature for half an hour.

When submitted to the above test the true dysenteric bacilli showed no macroscopic agglutination; all other members of the dysentery group (B. ambiguus, B. alkalescens, B. dispar, and a few others unplaced) were without exception vigorously agglutinated at some point in the six tubes, usually in the last 5, sometimes in all 6.

*Agglutination with Specific Sera.* All strains were tested against the various stock sera available and the following general conclusions appeared to result :

1. Shiga's bacillus is readily agglutinated to end-titer by any of the stock Shiga sera.

2. B. ambiguus is not agglutinated by Shiga sera, even in low dilution.

3. Flexner-Y bacilli are usually agglutinated to end-titer, or its neighbourhood, by one or another of the stock sera, but different strains may respond to different sera.

4. B. alkalescens fails to agglutinate with Flexner-Y sera within the usual time limit of 4 to 5 hours.

5. B. dispar is in much the same case.

The author concludes by a plea for careful observations on the agglutinating power of the patient's serum upon the organism isolated in suspected dysentery, as he considers this to be an important link in the chain of evidence.

